# Identifying Existing Evidence to Potentially Develop a Machine Learning Diagnostic Algorithm for Cough in Primary Care Settings: Scoping Review

**DOI:** 10.2196/46929

**Published:** 2023-12-14

**Authors:** Julia Cummerow, Christin Wienecke, Nicola Engler, Philip Marahrens, Philipp Gruening, Jost Steinhäuser

**Affiliations:** 1 Institute of Family Medicine University Medical Centre Schleswig-Holstein, Campus Lübeck Lübeck Germany; 2 Institute for Neuro- and Bioinformatics University of Lübeck Lübeck Germany

**Keywords:** cough, predictor, differential diagnosis, primary health care, artificial intelligence

## Abstract

**Background:**

Primary care is known to be one of the most complex health care settings because of the high number of theoretically possible diagnoses. Therefore, the process of clinical decision-making in primary care includes complex analytical and nonanalytical factors such as gut feelings and dealing with uncertainties. Artificial intelligence is also mandated to offer support in finding valid diagnoses. Nevertheless, to translate some aspects of what occurs during a consultation into a machine-based diagnostic algorithm, the probabilities for the underlying diagnoses (odds ratios) need to be determined.

**Objective:**

Cough is one of the most common reasons for a consultation in general practice, the core discipline in primary care. The aim of this scoping review was to identify the available data on cough as a predictor of various diagnoses encountered in general practice. In the context of an ongoing project, we reflect on this database as a possible basis for a machine-based diagnostic algorithm. Furthermore, we discuss the applicability of such an algorithm against the background of the specifics of general practice.

**Methods:**

The PubMed, Scopus, Web of Science, and Cochrane Library databases were searched with defined search terms, supplemented by the search for gray literature via the German *Journal of Family Medicine* until April 20, 2023. The inclusion criterion was the explicit analysis of cough as a predictor of any conceivable disease. Exclusion criteria were articles that did not provide original study results, articles in languages other than English or German, and articles that did not mention cough as a diagnostic predictor.

**Results:**

In total, 1458 records were identified for screening, of which 35 articles met our inclusion criteria. Most of the results (11/35, 31%) were found for chronic obstructive pulmonary disease. The others were distributed among the diagnoses of asthma or unspecified obstructive airway disease, various infectious diseases, bronchogenic carcinoma, dyspepsia or gastroesophageal reflux disease, and adverse effects of angiotensin-converting enzyme inhibitors. Positive odds ratios were found for cough as a predictor of chronic obstructive pulmonary disease, influenza, COVID-19 infections, and bronchial carcinoma, whereas the results for cough as a predictor of asthma and other nonspecified obstructive airway diseases were inconsistent.

**Conclusions:**

Reliable data on cough as a predictor of various diagnoses encountered in general practice are scarce. The example of cough does not provide a sufficient database to contribute odds to a machine learning–based diagnostic algorithm in a meaningful way.

## Introduction

Primary care is known to offer high-quality care, which, among others, has the most beneficial impact on mortality and costs [1]. The core discipline of primary care in Germany is general practice. General practice is one of the most complex medical specialties, as multiple reasons for encounters, diagnoses, and diagnostic procedures must be considered and mastered by integrating biopsychosocial aspects [2].

Thus, consultation in general practice represents a complex interaction between the patient and physician, including the parallelism between analytical and relational modes, such as pretest probability, gut feelings, and dealing with uncertainties [3]. Technical developments promise support for daily work processes, including the implementation of electronic health records [4]. Simultaneously, various methods of artificial intelligence (AI), such as machine learning and clinical decision support systems, are a developing field in future primary care [5,6].

Knowledge-based AI supplies expert opinions about the best patient care as an electronic database whereas a subset of data*-*based AI is machine learning, in which a computer learns to recognize the features in data, improving independently without the requirement to program a solution path. This enables the comparison of a particular patient with a model that has been previously trained on many patient cases by identifying patterns and relationships between data and outcomes of interest [7,8]. The benefits of AI can be seen in the sharing of large amounts of information between health care providers and researchers. Problems include the insufficient compatibility of systems (fragmentation), data security, the lack of traceability of an AI-based diagnosis (“black box effect”), the risk of hidden bias (eg, machine learning based only on data from certain homogeneous groups), and the risk of ignoring the influence of, for example, prevalence [9,10]. The potential of using AI is currently seen primarily in diagnostic imaging in medical specialties such as pathology, radiology, dermatology, and ophthalmology. Machine learning algorithms already offer numerous potential applications in this area [11,12].

The Medical Cause and Effect Analysis project at the University of Lübeck aims to develop a perspective for the use of AI in different sectors of health care. The project examines the extent to which existing software systems for modeling cause-effect relationships, as already used for analyzing complex technical systems, are suitable as a core for medical expert systems. The failure mode and effect analysis method is widely used in the automotive, aerospace, and medical technology industries [13]. To include general practice, the Institute of Family Medicine participated in this project by modeling the use case *cough*.

With a quite high prevalence (eg, 9.6% worldwide just for chronic cough) [14], cough is one of the most common reasons for a consultation in general practice [15,16]. The particular challenge for general practitioners (GPs) is to distinguish patients with harmless causes of cough from those in whom cough is the sign of a serious disease or even a potentially life-threatening diagnosis [17]. Red flags include respiratory, cardiovascular, constitutional, gastrointestinal, musculoskeletal, and psychosocial symptoms [18-20]. To translate the aspects of what happens during a consultation (eg, whether this patient appears to be seriously ill), probabilities for the underlying diagnoses (odds ratios [ORs]) would need to be determined first.

This requires some preliminary consideration of how AI has been used to date in the context of cough in general practice. For example, in respiratory medicine, there are efforts to develop an algorithm to support the interpretation of pulmonary function tests for the diagnosis of a range of obstructive and restrictive lung diseases [8]. Other AI applications use patient input in a symptom checker (including the symptom cough) to identify likely diagnoses and provide recommendations for the interval at which a physician should be consulted in person [21]. Furthermore, there are efforts to use cough acoustics for diagnostic algorithms, as cough contains information for numerous respiratory conditions [22]. Nevertheless, using cough acoustics is currently still limited, such as in studies of therapeutic success in chronic cough, infectivity in tuberculosis, and objectification of improvement in exacerbated chronic obstructive pulmonary disease (COPD) [23].

Thus, there are already various approaches to using the cough symptom for the medical use of AI. Nevertheless, the question remains as to what conclusions can be safely drawn from a patient presenting with a cough and whether the above-mentioned demands in the complexity of the primary care setting can be satisfactorily represented by AI-supported treatment pathways. In this context, it should be mentioned that the performance of data-driven diagnostic algorithms is dependent on the database used to train the machine learning model. Although it is desirable that these algorithms work with variable labels of data quality, their development and training require large volumes of well-structured data [8].

Thus, the first aim of this scoping review was to analyze the probability of a diagnosis based on the cough symptom in primary care. Therefore, in this paper, we identified studies that report on the existing data on cough as a predictor of several diagnoses encountered in general practice. The second goal was to subsequently reflect on whether these data are a suitable base to be used for training machine learning models related to the specifics of this clinical setting.

## Methods

To provide a transparent report of the results, we followed the guidelines of the PRISMA-ScR (Preferred Reporting Items for Systematic Reviews and Meta-Analyses extension for Scoping Reviews [24]; refer to Multimedia Appendix 1). A study protocol was not registered.

### Literature Search

The search was conducted until April 20, 2023. Two independent authors searched the PubMed, Scopus, and Web of Science databases for studies that provided information about cough as a predictor of any conceivable disease. The Cochrane Library was searched for additional data, supplemented by a search for gray literature in the web-based database of the German *Journal of Family Medicine*. To detect all relevant areas, a complex search strategy was developed, using not only the term “Cough,” but also terms presumed to be associated with cough. These were “Pulmonary Disease, Chronic Obstructive,” “Asthma,” “Pneumonia,” “Respiratory Tract Infections,” “Carcinoma, Bronchogenic,” “Gastroesophageal Reflux,” “Heart Failure,” and “Angiotensin-Converting Enzyme Inhibitors.” To identify studies reporting on cough as a predictor, we added the terms “predict*,” “Odds Ratio,” and “Likelihood Functions.” The terms “Family Practice” and “General Practice” were included to ensure relevance to primary health care. To exclude studies focusing on therapeutic issues, the search was combined with the term NOT “Therapeutics.” As far as possible, Medical Subject Headings terms were used. The start date for the search set was 1995 because this is the first year from which studies were available in sufficient volume. The complete search strategy is available in Multimedia Appendix 2. A total of 2 identified experts in the field of cough were contacted in April 2022 and asked about unpublished or ongoing studies.

### Study Selection

**Figure 1:**
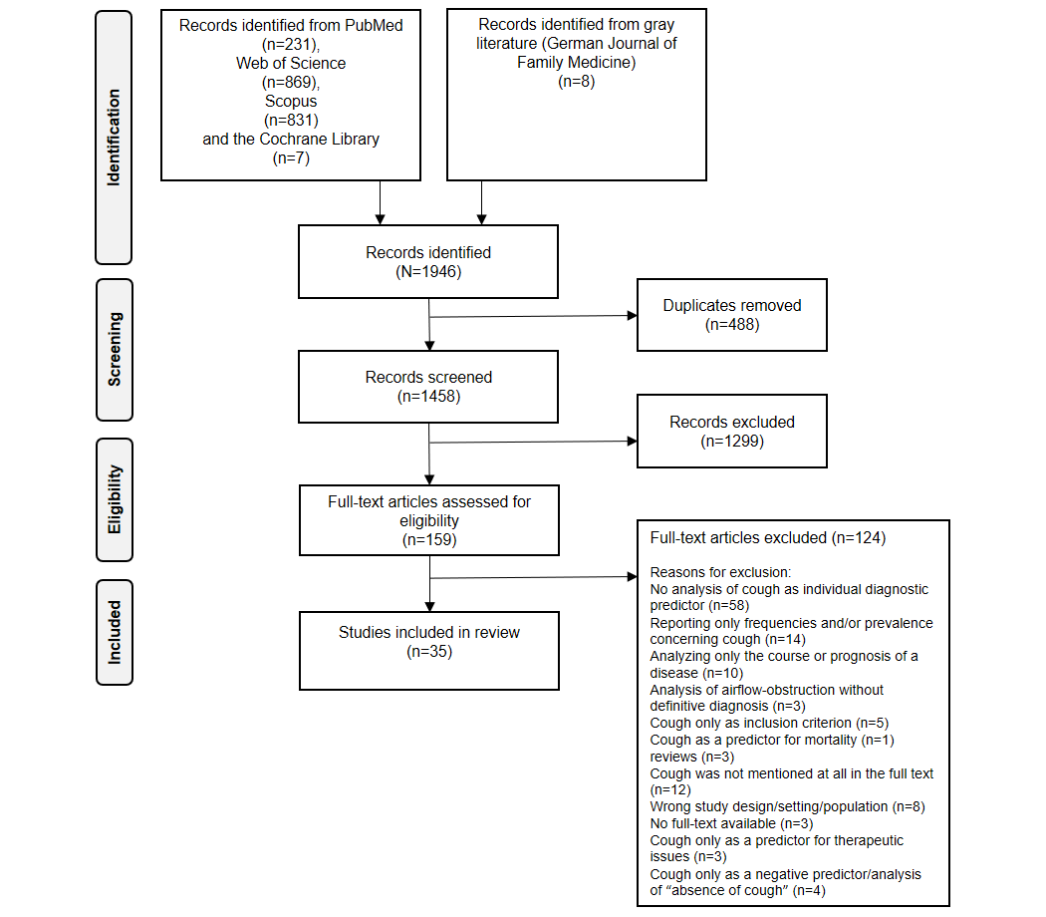
PRISMA (Preferred Reporting Items for Systematic Reviews and Meta-Analyses) flowchart. Visualization of the literature search, the screening process and selection of the studies included.

After the exclusion of duplicates, titles and abstracts of the search results were screened independently by 2 reviewers to exclude clearly irrelevant articles. Then a full-text analysis was performed to identify all relevant articles. Discrepancies during the screening process were discussed at all stages at regular consensus meetings. A third experienced reviewer was consulted to resolve disagreements. The entire screening process was visualized using the PRISMA flowchart, as presented in Figure 1 (adaptation from the study by Page et al [25]).

### Eligibility Criteria

We included all studies that reported data with diagnostic value related to cough. More precisely, only studies reporting on cough-related ORs, hazard ratios (HRs), positive predictive values (PPVs), negative predictive values (NPVs), and positive and negative likelihood ratios (LRs; LR+/LR-) were considered. Another inclusion criterion was for the study to have taken place in a primary care setting. We did not consider any therapeutic issues or studies reporting only the prevalence of cough. Further exclusion criteria were languages other than English or German and articles other than original studies (ie, reviews, letters, and case reports).

### Data Extraction and Analysis

The extraction of valid data was conducted by 2 reviewers (NE and JC), if necessary, under the supervision of an experienced third reviewer (JS). Data extraction included authors and year, study design, study population, country, and main results concerning ORs, (HRs), PPVs, NPVs, and LR+/LR-. The results were presented in tabular form and sorted thematically by diseases.

## Results

### Literature Search Results

The search from 1995 up until April 20, 2023, yielded 231 records from PubMed, 869 from Web of Science, and 831 from Scopus. In total, 7 articles were identified from the search of the Cochrane Library, and the search for gray literature in the German *Journal of Family Medicine* yielded 8 additional results. Of these 1946 results, 488 records were removed because of duplication (Figure 1). Title and abstract screening of the remaining 1458 records led to the exclusion of another 1299 articles. The remaining 159 articles were examined using a full-text inductive thematic analysis. The reasons for exclusion of 124 articles after full-text screening are presented in the PRISMA flowchart (Figure 1). Finally, a total of 35 articles were identified to meet the inclusion criteria. The 2 experts contacted were not aware of any additional unpublished or ongoing studies.

The included articles (for summarized results related to cough, refer to Multimedia Appendix 3 [26-60]) covered the following diagnoses: COPD, asthma, obstructive airway disease (OAD), influenza or influenza-like illness, respiratory tract infection (RTI), bronchial carcinoma (BC), community-acquired pneumonia, COVID-19, dyspepsia, gastroesophageal reflux disease (GERD), differential diagnosis of pulmonary embolism, heart failure, and adverse effects of angiotensin-converting enzyme (ACE) inhibitors. Of these studies, 10 were prospective studies (Vandevoorde et al [27], Freeman et al [30], Price et al [34], Eysink et al [38], Kable et al [39], Navarro-Marí et al [44], Thursky et al [45], Senn et al [46], Govaert et al [26], and Hollenz et al [59]), 8 were prospective cohort or diagnostic studies (Hamers et al [29], Schneider et al [37], Pescatore et al [40], Buffels et al [42], Schneider et al [53], Hippisley-Cox et al [54], Erkens et al [57], and Wallander et al [58]), 5 were cross-sectional studies (van Schayck et al [33], Melbye et al [36], Schneider et al [43], Kool et al [49], Hopstaken et al [52]), 4 were case-control studies (Haroon et al [35], Hamilton et al [55], Iyen-Omofoman et al [56], and Visser et al [60]), 2 were multicenter studies (Vrijhoef et al [32] and van Elden et al [47]), and 1 each was a descriptive (Thiadens et al [41]), retrospective (Nakanishi et al [51]), cohort (Geijer et al [28]), prospective case series (Bloom et al [50]), prospective, systematic sampling study (Sočan et al [48]) and comparative study (van Schayck et al [31]). Of these, 1 study included a secondary analysis of the data (Erkens et al [57]). Another study was listed as a randomized controlled trial, whereas the results listed here are considered prospective [26].

The number of patients varied widely; the largest study had >3 million patients, 5 included more than 5000 patients, another 5 had more than 1000 patients, 22 had between 100 and 1000 patients, and 3 had ≤100 patients. In total, 12 studies were conducted in the Netherlands; 7 in the United Kingdom; 4 in Germany; 2 each in Belgium and Australia; 1 each in Brazil, Japan, Norway, Slovenia, Spain, and Switzerland; and 1 combined in the United Kingdom and the United States. An overview of all the included studies is presented in Multimedia Appendix 3 [26-60].

### Topic-Specific Summary of Search Results

#### Obstructive Airway Diseases

A total of 11 articles were identified by referring to the topic of COPD. The variables “chronic cough,” “cough,” or “daily cough in periods” alone were identified as a predictor in 7 studies with ORs ranging from 1.46 (95% CI 0.75-2.84) to 4.4 (95% CI 2.8-6.7) [27,28,30,31,33,36,37]. Further associations were found for “weather affects cough” (OR 2.36; *P*=.04) and “coughing up phlegm without a cold” (OR 2.58; *P*=.002) [34]. A diagnosis of COPD was also associated with a more frequent number of primary care consultations with cough as the reason for the encounter (“presentations with cough, 1 episode within 3 years of COPD diagnosis”: OR 3.14, 95% CI 2.96-3.34; “>1 episode”: OR 7.12, 95% CI 6.64-7.63) [35]. “Morning cough,” in turn, did not predict COPD in 1 study [32], and in another, there was a small association to “coughing up phlegm in the morning” (OR 0.40; *P*=.01) [34]. Higher ORs than for cough alone in some studies were found for the symptom complexes “cough and dyspnea” (OR 3.41, 95% CI 1.31-8.90) [29] and, respectively, “chronic cough and phlegm” (OR 3.6), remaining significant in multivariate analysis (OR 1.7) [31], whereas the combination of “cough” and “age” did not reliably predict mild COPD [28].

There were 3 studies covering the diagnosis of asthma in children [38-40]. Only one of these studies reported an independent positive association of children’s asthma with the variable “night cough: more than 3 episodes in past 12 months” (OR 1.7; *P*=.04) [39]. “Exercise-related wheeze/cough” (OR 1.26) and “aeroallergen-related wheeze/cough” (OR 1.22) were identified to predict the diagnosis of asthma after 5 years in symptomatic children aged 1 to 3 years [40]. The third study did not show any significant association between cough and allergic asthma in children [38]. Another study showed a negative association between asthma and coughing in general practice (OR 0.36, 95% CI 0.20-0.63). The associated LRs increased for the complex of “no coughing,” “no history of smoking,” and “dyspnea attacks” (LR 4.08, 1.67-10.4) [37].

A total of 3 articles were identified concerning OADs that were not further specified. A study showed a significant association between cough and previous consultations because of wheezing and cough (multiple regression analysis: β=–.143, SE .030; standardized β=–.154; *t*=4.766; *P*<.001) [42]. Another study found a negative association between cough and OAD (OR 0.2, 95% CI 0.1-0.7; PPV 42.3%; NPV 23.5%) [43]. A third study addressed asthma and COPD (without differentiation) in adults without demonstrating a significant association with “night cough” [41].

#### Infectious Diseases

In total, 6 studies referred to influenza [26,44-48]. All of them identified some significant correlation of “cough” with influenza infection (OR 1.76, 95% CI 1.28-2.42 to 6.3, 95% CI 2.3-17.1; relative risk 11.7, 95% CI 1.40-97.5), although this was not the case for all years in all regions [45]. In total, 3 studies found that a combination of cough and elevated temperature raised the OR compared with looking at the variables individually (OR 2.24; OR 2.24, 95% CI 1.44-3.50 to 5.68, 95% CI 4.24-10.20; PPV 26.3). The additional consideration of an acute onset of symptoms strengthened the association between symptom complex and influenza infection. Thus, for the complex of “fever, coughing, and acute onset” the OR increased further (OR 7.87, 95% CI 4.96-12.50) [26,44,46]. The combination of “cough,” “headache at onset,” “feverishness at onset,” and “vaccination status in a period with increased influenza activity” showed the highest reported PPV of 75% [47], whereas cough alone had a PPV of 54% and NPV of 73%. Another study showed that the models and their values varied by year of influenza infection and by region; in one region, the combination of “cough, fever, fatigue, and myalgia” yielded the highest PPV (26.7%-47.4% dependent on the year), and in another region, it was “cough, fever, and fatigue” (PPV 59.7%) [45]. Differences were also found between children and adults (cough predicted influenza infection in adults both in univariate and in multivariate analysis and in children only in multivariate analysis) [48].

A total of 2 studies were identified as dealing with RTIs [49,50]. Cough predicted respiratory virus infection (univariable: OR 2.5, 95% CI 1.4-4.4; multivariable: OR 2.4, 1.3-4.3). In multivariate regression analysis, cough was not a significant predictor of adenovirus and respiratory syncytial virus infection [49]. Furthermore, no association of cough with a positive culture for bacterial pathogens of upper RTI was found [50].

In 1 study, “cough <2 days” univariably predicted pneumonia (OR 3.8, 95% CI 1.0-13.8, *P*<.05, PPV 36.4, NPV 86.9), and in multivariable analysis, the variables “dry cough” (OR 2.77, 95% CI 1.19-6.44), “diarrhea” (OR 5.90, 95% CI 1.89-8.49), and “temperature >38 °C” (OR 3.08, 95% CI 1.35-7.02) were statistically significant predictors of pneumonia [52]. In another study, cough did not predict community-acquired pneumonia among patients presenting with symptoms of lower RTI [51]. As far as COVID-19 is concerned, a significant association was found for the variable “dry cough” in patients with COVID-19 (OR 1.69, 95% CI 1.08-2.62). The PPV was 21%; the NPV was 86% [53].

#### Other Entities

The studies identified within this work that covered the diagnosis of BC reported that HRs, or, respectively, ORs, concerning hemoptysis exceeded those concerning cough (dependent on the study design and the considered interval in relation to the diagnosis). For example, multivariate analysis of the primary care records 2 years before diagnosis showed much higher values for “hemoptysis” (OR 32, 95% CI 13-81, *P*<.001) than for “second attendance with cough” (OR 2.7, 95% CI 1.7-4.4, *P*<.001) [54-56]. Another study analyzing the relevant differential diagnosis of pulmonary embolism also found higher associations of “hemoptysis” with clinically relevant diseases (ie, pneumonia, asthma or COPD, RTI, heart failure, pericarditis, lung cancer [OR 3.3, 95% CI 1.2-9.0]) than for “unexplained cough” (OR 2.0, 95% CI 1.3-3.0) [57].

Patients with (newly diagnosed) cough had an OR of 1.5, 95% CI 1.3-1.7 for being diagnosed with dyspepsia [58]. For the diagnosis of GERD, the OR was 3.7, 95% CI 1.7-7.6 [59].

One study investigated the association of cough and heart failure. No significant association of “daily cough in periods” and heart failure was found [36].

Although incident coughing was associated with ACE inhibitor use (OR 2.1, 95% CI 1.5-3.1), the adjusted results were not significant. Among the individual agents, only enalapril provided statistically significant results, both unadjusted (OR 2.6, 95% CI 1.6-4.2) and adjusted (OR 1.7, 95% CI 1.03-2.8), whereas the results for captopril, lisinopril, and perindopril were not statistically significant [60].

## Discussion

### Principal Findings and Comparison With Prior Work

In this scoping review, we included 35 studies reporting on cough as a predictor of different diagnoses in general practice. Most articles were found on obstructive airway diseases, followed by respiratory tract infections. Within these topics, symptom complexes yielded higher ORs than cough as a single symptom. A total of 3 articles were found on BC. Other entities were identified only in 1 article, respectively. Overall, data for diagnosis based on the symptom of cough are scarce. Comparisons of the studies have limitations because of the varying study sizes and designs.

The ORs for cough as a predictor of COPD are heterogeneous [27,28,30,31,33,36,37]. Overall, the study results suggest that cough is a relevant predictor of COPD, although only to a small extent as a single symptom. The combination of symptoms such as “cough and dyspnea” and “chronic cough and phlegm,” in turn, led to higher ORs through further specification [29,31]. Nevertheless, other authors report age and spirometry predicting COPD rather than the detection of clinical symptoms [61]. A relevance of the symptom cough for the diagnosis of chronic obstructive airway diseases other than COPD (ie, asthma and OAD) cannot be inferred from the inconsistent results of the rather small studies presented here [37,39-43]. Others report a stronger association of clinical symptoms and asthma [61]. However, they highlight wheezing and thoracic tightness, not cough, as the strongest predictors.

The results of cough as a predictor of infectious diseases are more consistent, although only a few studies are involved in each case. A significant correlation of influenza infection with “cough” was shown by all included studies on this topic, albeit with varying strengths of the associations [26,44-48]. The association was again enhanced by the combination of symptoms (here “cough and fever” and “fever, coughing, and acute onset”) [26,44,46]. This is in line with the situation in practice, where usually more than 1 symptom is considered for the diagnosis. Nevertheless, the predictive value might vary with years and the region where the infection occurs, as well as between children and adults [45,48] This could be related to different virus variants, for example. Only 1 included study reported on the COVID-19 infection [53]. Although it did predict the COVID-19-infection, a described clinical decision rule did not include cough. Others also stated a low predictive value of cough, fever, and headache for the diagnosis of the COVID-19-infection [62].

The included studies do provide evidence of associations of hemoptysis and serious disease (especially BC) [54-57], as well as a stronger association of cough and GERD [59] compared with cough and dyspepsia [58]. Nevertheless, the small number of results requires confirmation by further studies. Among ACE inhibitors, only enalapril was shown to be statistically significantly associated with incident cough [60]. However, this refers to only 1 study and therefore does not allow a reliable statement.

### Usability of the Results for AI

In summary, although cough is one of the most frequent reasons for seeing a GP, it is surprising how few studies on cough as a predictor of various diseases were identified, which could serve as a database in a diagnostic algorithm.

However, studies and reviews tend to start from the diagnosis (eg, pneumonia) [63,64] rather than from the symptoms or the reason for consultation. Thus, their conclusions are drawn backward from the diagnosis to the symptoms, as it were. Therefore, these studies represent an advanced diagnostic process with a level of uncertainty that is already reduced (“entropy”). To reach this stage in primary care, GPs actively reduce the uncertainties of an initially complex situation, meaning that the theoretically possible diagnoses have already been limited [65]. Accordingly, it is reported that analysis of the reason for encounters leads to lower PPVs than when using data documented by the GPs [66]. In contrast, although PPVs of reasons for encounter tend to be low overall, differences can still be noted. In a study in which the reasons for seeking primary medical care were analyzed in association with cancer diagnoses, the PPV of hemoptysis for cancer diagnoses was 2.7%, whereas cough as a reason for encounter yielded a PPV of 0.1% [66]. The chance of having pneumonia is reported to be higher in patients who presented with cough and fever than in those who reported only cough as the reason for encounter (16.4% vs 5.6%) [67]. A broader study base that considers this approach would be desirable as a starting point for probability calculations in primary care—not least in terms of its potential application in the context of AI. Furthermore, in the patient-centered setting of general practice with a wide array of possible diagnoses, the cognitive strategies used by clinicians often contain unusual phenomena (ie, symptoms, pattern failure, and a sense of alarm) [68]. A lot of tacit information flows preconsciously into a diagnosis. This is not reflected in the studies analyzed in this paper. It seems questionable how this database could be used to translate the reality of primary care into a reliable machine-based diagnostic algorithm. Thus, as already assumed for internal medicine, general medicine may be an area where the implementation of AI-based algorithms is more complex than in disciplines with frequent use of diagnostic imaging [11]. Future research needs to always include the sector-adapted prevalence of a disease when looking at PPVs.

### Limitations

Our search strategy aimed to identify the broadest possible range of relevant articles. Nevertheless, the search focused on studies written in English or German. Thus, we might have missed studies written in other languages. The wide range of numbers and ages among the participants limits the comparability of the study results. The largest study used a dedicated database for research in the United Kingdom based on physicians’ routine documentation. Thus, even in this large study, a bias because of differences in quality and missing documentation must be considered [54]. In addition, the focus on primary care might have led to an underrepresentation of severe acute illnesses, as studies reporting these conditions may be more likely to be conducted in a hospital setting. Nevertheless, some studies reported different results in different settings [37,69]. Therefore, the results of studies in a hospital setting need to be transferred with caution to general practice and vice versa.

### Conclusions

Although the symptom of cough is one of the most frequent reasons for consultation in general practice, data for probabilities of the underlying causes are scarce. The complex processes that occur in general practice on the path from naming the reason for the consultation to finding the diagnosis are still underrepresented in studies that include cough as a predictor of various diseases. This should be considered when planning the development of machine-based diagnostic algorithms for general practice. Until solid evidence is available in this field, it seems questionable whether AI solutions can be programmed to adequately reflect reality.

## Appendix

Multimedia Appendix 1 PRISMA-ScR (Preferred Reporting Items for Systematic Reviews and Meta-Analyses extension for Scoping Reviews) checklist.

Multimedia Appendix 2 Search terms (presentation of the search terms used for the PubMed, the Cochrane Library, Web of Science, and Scopus databases and the German Journal of Family Medicine).

Multimedia Appendix 3 Summarized characteristics of included studies.

## Abbreviations

ACE: angiotensin-converting enzyme
AI: artificial intelligence
BC: bronchial carcinoma
COPD: chronic obstructive pulmonary disease
GERD: gastroesophageal reflux disease
GP: general practitioner
HR: hazard ratio
LR: likelihood ratio
NPV: negative predictive value
OAD: obstructive airway disease
OR: odds ratio
PPV: positive predictive value
PRISMA-ScR: Preferred Reporting Items for Systematic Reviews and Meta-Analyses extension for Scoping Reviews
RTI: respiratory tract infection
