# Supplementary material for: Identifying Existing Evidence to Potentially Develop a Machine Learning Diagnostic Algorithm for Cough in Primary Care Settings: Scoping Review
Source: J Med Internet Res. 2023 Dec 14;25:e46929. doi: 10.2196/46929 (PMC10755665; doi:10.2196/46929)
Supplement: Multimedia Appendix 2 [file jmir_v25i1e46929_app2.pdf]

Search terms

Searchterm PubMed

(("Family Practice"[Mesh] OR "General Practice"[Mesh]) AND ("Cough"[Mesh] OR "Pulmonary Disease, Chronic Obstructive"[Mesh] OR "Asthma"[Mesh] OR "Pneumonia"[Mesh] OR "Respiratory Tract Infections"[Mesh] OR "Carcinoma, Bronchogenic"[Mesh] OR "Gastroesophageal Reflux"[Mesh] OR "Heart Failure"[Mesh] OR "Angiotensin-Converting Enzyme Inhibitors"[Mesh]) AND ("predict*" OR "Odds Ratio"[Mesh] OR "Likelihood Functions"[Mesh])) NOT Therapeutics"[Mesh])

Searchterm Cochrane Library

(("Family Practice"[Mesh] OR "General Practice"[Mesh]) AND ("Cough"[Mesh] OR "Pulmonary Disease, Chronic Obstructive"[Mesh] OR "Asthma"[Mesh] OR "Pneumonia"[Mesh] OR "Respiratory Tract Infections"[Mesh] OR "Carcinoma, Bronchogenic"[Mesh] OR "Gastroesophageal Reflux"[Mesh] OR "Heart Failure"[Mesh] OR "Angiotensin-Converting Enzyme Inhibitors"[Mesh]) AND ("predict" OR "Odds Ratio"[Mesh] OR "Likelihood Functions"[Mesh])) NOT Therapeutics"[Mesh])

Searchterms adapted for the German Journal of Family Medicine

“Husten“ [cough], „COPD“, „Asthma“, „Pneumonie“ [pneumonia], „Atemwegsinfekt“ [respiratory tract infection], „Bronchialkarzinom“ [bronchogenic carcinoma], „gastroösophagealer Reflux“ [gastroesophageal reflux disease], „Herzinsuffizienz“ [heart failure]

Searchterm Web of science

((TS=(Family Practice) OR TS=(General Practice)) AND (TS=(Cough) OR TS=(Chronic Obstructive Pulmonary Disease) OR TS=(Asthma) OR TS=(Pneumonia) OR TS=(Respiratory Tract Infections) OR TS=(Bronchogenic Carcinoma) OR TS=(Gastroesophageal Reflux) OR TS=(Heart Failure) OR TS=(Angiotensin-Converting Enzyme Inhibitors)) AND (TS=(predict) OR TS=(Odds Ratio) OR TS=(Likelihood Functions))) NOT TS=(Therapeutics) NOT TS=(Therapy)
(Index date 1.1.1995-20.04.2023)

Searchterm Scopus

(((TITLE-ABS-KEY ("Family Practice") OR TITLE-ABS-KEY ("General Practice")) AND (TITLE-ABS-KEY ("Cough") OR TITLE-ABS-KEY ("Chronic Obstructive Pulmonary Disease") OR TITLE-ABS-KEY ("Asthma") OR TITLE-ABS-KEY ("Pneumonia") OR TITLE-ABS-KEY ("Respiratory Tract Infection*") OR TITLE-ABS-KEY ("Bronchogenic Carcinoma") OR TITLE-ABS-KEY ("Gastroesophageal Reflux") OR TITLE-ABS-KEY ("Heart Failure") OR TITLE-ABS-KEY ("Angiotensin Converting Enzyme Inhibitor*")) AND (TITLE-ABS-KEY ("predict*") OR TITLE-ABS-KEY ("Odds Ratio") OR TITLE-ABS-KEY ("Likelihood Function*"))) AND NOT TITLE-ABS-KEY ("Therapeutics") AND NOT TITLE-ABS-KEY ("Therapy")) AND PUBYEAR > 1994
