# Supplementary material for: Identifying Existing Evidence to Potentially Develop a Machine Learning Diagnostic Algorithm for Cough in Primary Care Settings: Scoping Review
Source: J Med Internet Res. 2023 Dec 14;25:e46929. doi: 10.2196/46929 (PMC10755665; doi:10.2196/46929)
Supplement: Multimedia Appendix 3 [file jmir_v25i1e46929_app3.pdf]

Table 1. Summarized characteristics of included studies.

Study	Country	Design	Study sample	Diagnosis	Main results in terms of cough
Vandevoorde et al [27], 2007	Belgium	Prospective study	146 active smokers with ≥ 15 pack-years (38% female), 40-70 y; 8 GPs[a] in 6 practices	COPD[b]	In univariate analysis the OR[c] of having COPD were influenced by "age" (OR 1.04, 95% CI 1.00-1.08, *P*=.038), "chronic cough" (OR 2.29, 95% CI 1.18-4.44, *P*=.015), "subjective wheezing" (OR 2.71, 95% CI 1.36-5.41, *P*=.005), and "objective wheezing" (OR 5.19, 95% CI 2.38-11.28, *P*<.001). In the multivariate analysis only "objective wheezing" remained an independent predictor (OR 4.72, 95% CI 2.14-10.37, *P*<.001). Comparing newly detected patients with COPD with known patients with COPD in the univariate analyses, patients were more likely to have COPD diagnosed if they were "older," had a "history of more pack-years," and the symptoms "fatigue," "dyspnea on exertion," "chronic cough" and "expectorations." In the multivariate analysis, only "age" (OR 1.12, 95% CI 1.04-1.21, *P*=.003), "chronic cough" (OR 7.64, 95% CI 1.87-31.18, *P*=.005), and "fatigue" (OR 3.99, 95% CI 1.13-14.08, *P*=.032) remained significant.

Geijer et al [28], 2006	Netherlands	Cohort study	567 male current smokers without known lung-disease (no female), 40-65 y, primary care	COPD (early detection)	In the univariate analysis “age ≥55 y” (OR 3.2, 95% CI 1.9-5.6), “pack-years ≥30” (OR 3.0, 95% CI 1.8-5.0), and “cough” (OR 2.4, 95% CI 1.5-3.8) predicted mild COPD. In the multivariate analysis only “age” and “cough” (“cough”: OR 2.3, 95% CI 1.5-3.7; “age” (years): OR 1.4, 95% CI 1.2-1.7) were predictors of mild COPD. The combination of these variables did not satisfactorily predict the presence of mild COPD (ROC[d] area 0.65, 95% CI 0.59-0.70).
Hamers et al [29], 2006	Brazil	Prospective cohort study	350 patients (≥15 y) presenting with shortness of breath and/or cough, 157 identified for spirometry, thereof 142 valid spirometries (49% male), 34 GPs	COPD	In the multivariate analysis independent predictors for spirometric diagnosis of COPD were “age ≥55 y” (OR 8.62, 95% CI 1.87-39.7; PPV[e] 37.2%) and the symptom complex “dyspnea and cough” (OR 3.41, 95% CI 1.31-8.90; PPV 32.3%; each symptom alone was not significant). In the bivariate analyses, significant results are also shown for “ever smokers” (OR 4.64, 95% CI 1.78-12.1; PPV 35.3%), “smoking history ≥5 pack-years” (OR 8.65, 95% CI 3.04-24.7; PPV 45.9%), and “male sex” (OR 2.71, 95% CI 1.23-5.99; PPV 34.8%).

Freeman et al [30], 2005	United Kingdom	Prospective study	369 patients aged ≥40 y (52% male) with respiratory medications in previous 2 y, history of smoking or history of asthma without current medication. 1 primary care clinic.	COPD	Two questionnaire versions concerning "age," "smoking history," "cough," "dyspnea," and "wheeze" identified predictors of having COPD (ORs >1.0). In the version with shortened response categories, these were "age 55-69 y" (OR 4.6, 95% CI 1.3-16); "age ≥70 y" (OR 2.1, 95% CI 6.1-73); "cough occasional or more often" (OR 2.4, 95% CI 1.2-4.7); "dyspnea on any exercise or at rest" (OR 3.0, 95% CI 1.5-5.9); and "everyday wheeze" (OR 2.2, 95% CI 0.89-5.5). This questionnaire version identified COPD patients with a Sens[f] of 87.1% and Spec[g] of 71.3% (PPV 38.0%; NPV[h] 96.5%). A longer version with more response categories identified patients with COPD with a Sens of 77.4% and Spec of 76.2% (PPV 39.7%; NPV 94.4%). In this questionnaire version predictors of having COPD concerning cough were "cough only with exacerbations" (OR 3.1, 95% CI 0.98-9.9), "cough most mornings" (OR 2.2, 95% CI 0.75-6.2), and "cough every day" (OR 1.8, 95% CI 0.65-5.0).
van Schayck et al [31], 2005	Netherlands	Compara-tive study	Case-finding scenario to identify persons with COPD from a general population: 5030 patients (49.5% male; airway obstruction vs no obstruction);	COPD	Relevant variables significant (*P*<.001) in bivariate analysis for airway obstruction vs no obstruction: "age ≥70 y" (OR 1.5), "pack-years 24-29" (OR 2.1)/"≥50" (OR 3.2), "cough for 3 months" (OR 2.7), "chronic cough" (OR 3.1), "phlegm for 3 months" (OR 2.6), "chronic phlegm" (OR 2.9), "chronic cough or phlegm" (OR 2.9), "chronic cough and phlegm" (OR

			differential diagnosis scenario: 1018 patients (52% male; COPD vs asthma)		3.6), "cough or phlegm for 3 months" (OR 2.4), "cough and phlegm for 3 months" (OR 3.6), "dyspnea" (OR 1.8), "wheeze" (OR 2.8), "wheezing episodes (continuous)" (OR 2.4), and "wheeze no cold" (OR 2.9). Remaining significant in multivariate (range) analysis are "age ≥70 years" (OR 1.5), "pack-years ≥50" (OR 2.4-3.0), "chronic cough or phlegm" (OR 1.5), "chronic cough and phlegm" (OR 1.7), and "wheeze" (OR 1.5-1.6). Variables significant (*P*<.001) in bivariate analysis for airway obstruction vs asthma: "Pack-years ≥50" (OR 2.2), "cough for 3 months" (OR 2.4), "chronic cough" (OR 2.2), "phlegm for 3 months" (OR 2.7), "chronic phlegm" (OR 2.4), "chronic cough or phlegm" (OR 2.4), "chronic cough and phlegm" (OR 3.2), "cough or phlegm for 3 months" (OR 2.1), "cough and phlegm for 3 months" (OR 3.1), "wheeze" (OR 1.8), and "wheeze no cold" (OR 2.2). Only "pack-years ≥50" (OR 1.8-2.3) remained significant in multivariate (range) analysis. Ability of items to discriminate between airway obstruction vs no obstruction (Sens, Spec, PPV, and NPV): "age+chronic cough or phlegm" (54, 68, 21, and 91, respectively); "age+smoking status+pack-years+BMI" (64, 70, 25, and 93, respectively); "age+smoking status+pack-years+BMI+chronic cough or phlegm" (65, 70, 25, and 93, respectively); and "age+smoking status+pack-years+BMI+prior diagnosis (chronic

					bronchitis, emphysema, or asthma)+chronic cough or phlegm" (71, 67, 25, and 94, respectively). Ability of items to discriminate between airway obstruction vs asthma: "smoking status+pack-years+BMI" (63, 70, 69, and 64, respectively); "smoking status+pack-years+BMI+prior diagnosis+cough or phlegm for 3 months" (75, 63, 68, and 70, respectively); "smoking status+pack-years+BMI+prior diagnosis+cough or phlegm for 3 months+wheeze no cold" (73, 63, 68, and 69, respectively); and "smoking status+pack-years+BMI+prior diagnosis+cough or phlegm for 3 months+wheeze no cold+age" (72, 64, 68, and 68, respectively).
Vrijhoef et al [32], 2003	Netherlands	Multicenter study	Assessment of respiratory function of 231 smokers (>10 pack-years) aged 40-70 y (48.5% male), consulting reasons unrelated to respiratory diseases, 8 GPs	COPD	"Morning cough" did not show any significant results. Prevalence of "morning cough" with normal airflow (25%; n=162) and with limited airflow (37%; n=43; univariate: OR 1.81, 95% CI 0.36-3.81; multivariate: OR 0.95, 95% CI 0.48-8.28). Prevalence of "morning cough" without COPD (26%; n=189) and with COPD (44%; n=16; univariate: OR 2.22, 95% CI 0.79-6.28; multivariate: OR 0.78, 95% CI 0.12-5.31).
van Schayck et al [33], 2002	Netherlands	Cross-sectional study	Random selection of 651 patients (62% female), thereof identification of 201 smokers without	COPD	"Cough" for airflow obstruction among smokers had a PPV of 27. "Chronic cough" was significantly related to reduced lung function (OR 2.50, 95% CI 1.14 - 5.52, NPV 87). For every smoker with cough found to be

			respiratory medication, aged 35-70 y, 2 GPs		at risk, about 4 smokers with cough had to be tested. The PPV of at least 2 of the symptoms (“cough,” “dyspnea,” or “wheeze”) was only slightly higher (PPV 29), implying there is little additive value in asking about symptoms other than “cough.” When “cough” was present, with increasing age there was an increased chance of detecting patients with obstruction. Smokers with “cough” >60 y had a 48% chance of having bronchial obstruction.
Price et al [34], 2006	United Kingdom and United States	Prospective study	818 current or former smokers aged ≥40 y without prior respiratory diagnosis/medication (49.3% male), recruitment took place via random selection from primary care practice rosters in 1 town in the United Kingdom and 1 town in the United States	COPD	“Weather affects cough” (OR 2.36; *P*=.042); “coughing up phlegm without a cold” (OR 2.58; *P*=.002), and coughing up “phlegm in the morning” (OR 0.40; *P*=.011); further predictors were age, pack-years, BMI, wheezing frequency, and history of any allergies.

Haroon et al [35], 2015	United Kingdom	Case-control study	One patient with newly diagnosed COPD matched to 2 controls (incidentally, matching age, sex, and GP); 15,159 patients with newly diagnosed COPD and 28,296 controls (mean age 70 y, 52% male) from 340 general practices	COPD	Presentation with cough was significantly more often for cases than controls (presentations with cough, 1 episode within 3 years of COPD diagnosis: OR 3.14, 95% CI 2.96-3.34, >1 episode: OR 7.12, 95% CI 6.64-7.63) Adjusted regression analysis showed a stronger association of "presentations with cough >1 episode within 3 years of COPD diagnosis" (OR 1.77, 95% CI 1.59-1.97) than "presentations with cough—1 episode" (OR 1.42, 95% CI 1.30-1.56). Further significant associations were seen for smoking status, asthma, number of LRTI[i], presentations with dyspnea, wheeze, sputum production, unintended weight loss, antibiotic courses for an LRTI, and prescriptions of Salbutamol and Prednisolone. The final model included history of smoking, asthma, salbutamol prescriptions, and number of LRTIs in the previous 3 years, not cough.

Melbye et al [36], 2020	Norway	Cross-sectional study; health survey in 1 Norwegian community	Invitation of all residents ≥ 40 y to participate a first visit; random invitation for a second visit; finally, 1538 (48.5% male) were evaluated for heart failure and COPD	COPD and heart failure	"Daily cough in periods" predicted COPD (univariable: OR 4.4, 95% CI 2.8-6.7; multivariable: OR 3.4, 95% CI 1.9-6.3). Further anamnestic predictors of COPD in multivariable analysis were current and previous smoking, more shortness of breath than normal on the examination day, and ever diagnosed asthma.<br>"Daily cough in periods" was no predictor of heart failure.
Schneider et al [37], 2012	Germany	Prospective diagnostic study	Investigation of different health care sectors, 219 patients thereof (58% female), 10 GPs	COPD and asthma	Diagnostic accuracy of clinical symptoms for COPD (95% CI) in different clinical settings—results for "coughing" in GP: Sens 65.3 (51.3-77.1), Spec. 43.7 (36.4-51.3), PPV 25.4 (18.6-33.7), NPV 81.1 (71.8-87.9), OR 1.46 (0.75-2.84), LR[j]+ 1.16 (0.91-1.48), and LR- 0.79 (0.52-1.21). Diagnostic accuracy of clinical symptoms for asthma (95% CI) in different clinical settings—results for "coughing" in GP: Sens 43.8 (34.0-54.2), Spec 31.5 (24.1-40.0), PPV 31.0 (23.5-39.5), NPV 44.4 (34.6-54.7), OR 0.36 (0.20-0.63), LR+ 0.64 (0.49-0.83), and LR- 1.78 (1.30-2.45).<br>Adjusted LR for patients from GP for having asthma (n= 219, factor present vs factor absent) were as follows: "no coughing" 1.6 (1.2-2.2) vs 0.5 (0.4-0.8), "dyspnea attacks"

					1.7 (1.2-2.4) vs 0.8 (0.7-0.9), "no smoking" 1.5 (1.2-2) vs 0.6 (0.5-0.8), and "all 3 factors combined" 4.08 (1.67-10.4) vs 0.24 (0.12-0.58).
Eysink et al [38], 2001	Netherlands	Prospective study (2-year follow-up)	162 children (49.4% male), 1-5 y, presenting with cough >5 days, no history of allergic reactions or IgE[k] positiveness 136 GPs	Allergic asthma	The univariate analysis (unadjusted and adjusted for family history of allergy, breastfeeding, and age at the time of the second blood sample) did not show any significant results for cough. Of the children presenting with recurrent coughing (≥6 visits), the children who tested IgE-positive had been diagnosed with asthma in 73% (8/11) of cases in contrast to 24% (28/119) of the IgE-negative children.
Kable et al [39], 2001	Australia	Prospective study	169 children (no gender information), 18 months—18 y, 5 group practices	Asthma	The variables "parent/self-reported asthma" (OR 15.3, *P*<.001), "previous diagnosis" (OR 2.4, *P*=.005), "wheeze in last 12 months" (OR 2.1, *P*=.003), "visited GPs more than 3 times in past 12 months" (OR 2.0, *P*=.02), "night cough: more than 3 episodes in past 12 months" (OR 1.7, *P*=.04), and "symptoms with physical activity" (OR 1.6, *P*=.04) were independently associated with asthma as assessed by a physician.
Pescatore et al [40], 2014	United Kingdom	Prospective cohort study (5-year follow-up)	1226 individuals aged 1-3 years (55% male) from a population-based cohort consulting for wheezing or cough;	Asthma	Variables identified to predict asthma in symptomatic children were male sex (OR 1.48), age >1 y (OR 1.19), wheezing without colds (OR 1.4), wheezing frequency (> 3 attacks; OR 1.65), activity disturbance (any: OR 1.28; a lot: OR 1.63), shortness of breath (sometimes or always: OR 1.98, always: OR

			assessment of asthma 5 y later		1.56), exercise-related wheezing/cough (OR 1.26) and aeroallergen-related wheezing/cough (OR 1.22), eczema (OR 1.52), and parental history of asthma/ bronchitis (1 parent: OR 1.23; both parents: OR 1.26). CIs for the ORs are not provided. 10or an explanation, see the study.
Thiadens et al [41], 1998	Netherlands	Descriptive study	192 patients (38% male), 18-75 y, coughing ≥2 weeks, 1 primary health care center with 6 GPs	Asthma and COPD (without differentiation)	"Nocturnal coughing over the past 2 weeks," did not reach significance, nor did "childhood symptoms" or a "family or personal history of atopy." Significant ORs were found for "wheezing" and "attacks of dyspnea over the past year" but not for "episodes of (nocturnal) coughing over the past year" (OR 0.8, 95% CI 0.4-1.6) or "current nocturnal coughing" (OR 1.4, 95% CI 0.7-2.5).
Buffels et al [42], 2004	Belgium	Prospective cohort study	Cohorts: 222 patients without complaints (49% male), 703 with complaints (44% male); patients aged 35-70 y, 22 GPs	OAD[l]	Regression model for the diagnostic value of different symptoms; the dependent variable is obstruction: "previous consulting GP because of wheezing/cough" was the most relevant item (β=-.143, SE=0.030; standardized β=-.154; *t*=4.766; *P*<.001). "Wheezing" and "breathing difficulty" as symptoms were also significant parameters, whereas "cough" alone was not.

Schneider et al [43], 2006	Germany	Cross-sectional diagnostic study	60 patients (67% female) presenting with dyspnea, coughing, expectoration, or self-reported wheezing, mean age 46 y (SD 18), 6 GPs	OAD	Significant association with the diagnosis of OAD was found for the cutoff of "CRP >2mg/dL" (OR 4.4, 95% CI 1.4-13.8) and "self-reported wheezing" (OR 3.4, 95% CI 1.1-10.3), whereas the association of "coughing" was negatively (OR 0.2, 95% CI 0.1-0.7). Further values for "coughing": Sens 29.7, 95% CI 17.5-45.8, Spec 34.8, 95% CI 18.8-55.1, PPV 42.3, 95% CI 25.5-61.1, NPV 23.5, 95% CI 12.4-40.0, LR+ 0.5, 95% CI 0.3-0.8, and LR- 2.0, 95% CI 1.1-3.7.
Navarro-Marí et al [44], 2005	Spain	Prospective study	1934 patients (51% male) aged 2 months—96 y, presenting with symptoms of acute respiratory infection (<72 h duration), 20 physicians (17 GPs and 3 pediatricians)	Influenza/ARTI[m]	Bivariate analysis demonstrated that for the "ICHPPC[n]-2 criteria" (OR 4.34, 95% CI 3.41-5.51), "fever" (OR 4.88, 95% CI 3.75-6.34), "cough" (OR 1.76, 95% CI 1.28-2.42), and "rhinorrhea" (OR 1.40, 95% CI 1.08-1.82) individually showed a positive correlation with the number of positive results while "vaccination" showed a negative correlation (OR 0.32, 95% CI 0.20-0.53). "Cough" was significant in patients aged <50 y (<14 and 15-49 y), the combination of "fever" and "cough" (OR 2.24, 95% CI 1.44-3.50) gave similar results to those obtained for "ICHPPC-2 criteria" (OR 2.71, 95% CI 2.08-3.52).
Thursky et al [45], 2003	Australia	Prospective study	Patients with defined symptoms (Western Australia: 328 [50% male] in 1998, 191 [48% male] in 1999; in Victoria: 128 [45%	Influenza (laboratory confirmed)	In univariate analysis for Western Australia in 1998, fever (OR 2.8, 95% CI 1.5-5.3, *P*=.001), fatigue (OR 3.4, 95% CI 1.8-6.3, *P*=.0001), cough (OR 6.3, 95% CI 2.3-17.1, *P*=.0001), and myalgia (OR 2.6, 95% CI 1.3-4.9, *P*=.005) were significant predictors of the diagnosis of

			male] combined in 1998/1999) from 11 general practices from Perth (Western Australia) in 1998 and 6 in 1999, 10 general practices from Melbourne (Victoria) in 1998 and 14 in 1999		influenza. For Western Australia in 1999, not cough but chills (OR 8.2, 95% CI 3.3-20.5, *P*=.0001) and fatigue (OR 2.6, 95% CI 1.1-6.2, *P*=.03) were significantly associated with the diagnosis of influenza, whereas in Victoria (combined detection 1998/1999), only cough (OR 4.5, 95% CI 1.3-15.9, *P*=.02) predicted influenza.<br>In multivariate analysis, cough (OR 7.7, 95% CI 2.3-25.6), fever (OR 2.3, 95% CI 1.1-4.6), and fatigue (OR 2.6, 95% CI 1.3-5.2) were independent positive predictors of laboratory-confirmed influenza (age and sex adjusted results) in Western Australia in 1998.<br>In Western Australia, the model with the maximum PPV was cough, fever, fatigue, and myalgia (in 1998: Sens 56.3, Spec 84.9, PPV 47.4% (41.9-52.8), NPV 88.9 (85.5-92.3); in 1999: Sens 34.8, Spec 86.9, PPV 26.7% (20.4-32.9), NPV 90.7 (86.6-94.8); in Victoria, the model with the highest PPV was cough, fever, and fatigue (Sens 75.1, Spec 46.6, PPV 59.7 [50.9-68.6], NPV 64.3 [55.7-72.9]).

Senn et al [46], 2005	Switzerland	Prospective study	201 patients (49% female) with GP-suspected influenza, 1 outpatient clinic (primary care center)	Influenza	"Temperature >37.8 °C," "cough," "duration of symptoms <48 h before consultation," and "myalgia" were associated with a diagnosis of influenza. Further results for "temperature >37.8 °C": 74% of patients with a positive swab, 40% with a negative swab, OR 4.2 (95% CI 2.3-7.7); "cough": 96% with a positive swab, 89% with a negative swab, OR 3.2 (95% CI 1.0-10.4); combination of "temperature > 37.8 °C" and "cough": 72% with a positive swab, 37% with a negative swab, OR 4.4 (95% CI 2.4-7.9). "Cough" had a PPV of 54, NPV of 73, Sens of 96, and Spec of 11 for the diagnosis of influenza. "Temperature >37.8 °C" had a PPV of 66, NPV of 68, Sens of 74, and Spec of 60. Combination of "cough and temperature >37.8 °C" had a PPV of 68, NPV of 68, Sens of 72, and Spec of 63.
van Elden et al [47], 2001	Netherlands	Multicenter question-naire study	81 febrile patients (41% male) with at least 1 constitutional and 1 respiratory symptom, 14 GPs	Influenza and ILI[o]	"Cough" was significantly correlated with influenza A virus infection, compared with a group of patients of which other respiratory viruses or no viral pathogen could be detected (*P*≤.01, PPV 57, NPV 90, RR 11.7 [95% CI 1.40-97.5]). A PPV of 75 and NPV of 80 was demonstrated for combination of "cough," "headache at onset," "feverishness at onset," and "vaccination status" during the "period with increased influenza activity."
Govaert et al [26], 1998	Netherlands	Prospective study	1828 patients ≥60 y (no gender	Influenza and ILI	"Coughing" was significantly associated with the serologically confirmed diagnosis

		(results from a randomized controlled trial concerned only vaccination, not the results reported here)	information), with influenza-like symptoms but not belonging to the high-risk group, 34 GPs in 15 practices		influenza (OR 6.2, 95% CI 4.15-9.23, PPV 17, NPV 97, Sens 66, and Spec 76). For the complex of "fever, coughing and acute onset," the OR increased (OR 7.87, 95% CI 4.96-12.50, PPV 30.3) and was lower for the symptom complexes "fever and acute onset" (OR 5.83, 95% CI 3.76-9.03, PPV 24.3), "fever and coughing" (OR 5.68, 95% CI 4.24-10.20, PPV 26.3), and "fever, coughing, acute onset and malaise" (OR 7.43, 95% CI 4.64-11.90, PPV 29.5). Multiple logistic regression including vaccination and risk status revealed that "coughing" (OR 5.25, 95% CI 3.28-8.39, *P*=.001) and "fever" (OR 2.18, 95% CI 1.33-3.57, *P*=.002) were positive predictors; "vaccination" (OR 0.56, 95% CI 0.36-0.86, *P*=.009) a negative predictor of serological confirmed influenza.
Sočan et al [48], 2010	Slovenia	Prospec-tive, systematic sampling study	476 children (50% male) and 515 adults (41.7% male) with symptoms of ILI from 32 primary care centers	Influenza and ILI	In univariate analysis, "cough" was a significant predictor of influenza for adults (OR 3.096, 95% CI 1.378-6.98; *P*=.006) but not for children (OR 1.450, 95% CI 0.710-2.959; *P*=.308); in multivariate analysis, for adults, fever ≥38 °C (OR 2.395, 95% CI 1.250-4.588, *P*=.008) and cough (OR 2.623, 95% CI 1.153-5.965, *P*=.021) remained positive predictors; the significance of abnormal breathing sounds was stated as marginal. The model is reported to have Sens of 0%, Spec of 100%, PPV 0%, and NPV 76.4%.

					For children, multivariate analysis showed statistical significance for the variables fever ≥38 °C (OR 6.823, 95% CI 1.581-29.448; *P*=.010), headache (OR 2.117, 95% CI 1.022-4.984; *P*=.030), cough (OR 2.257, 95% CI 1.022-4.984; *P*=.044), and the absence of abnormal breathing sounds (OR 0.254, 95% CI 0.084-0.762; *P*=.015), Sens 5.1%, Spec 98.1%, PPV 57.1%, and NPV 80.1%.
Kool et al [49], 2015	Netherlands	Cross-sectional study	257 febrile children (41.2% female) aged 3 mo-6 y presented at a GP out of hours service, nasopharyngeal swabs taken by a research nurse, 1 GP out of hours service	RTI[p]	Cough (absence of a virus versus at least one virus present: OR 2.5 (95% CI 1.4-4.4) and temperature ≥38.0 °C (OR 2.1, 95% CI 1.3-3.5) predicted respiratory virus infection (positive nasopharyngeal swab for adenovirus, human bocavirus, enterovirus, human COVID-19 types OC-43, 229E, NL63 and HKU, human metapneumovirus, influenza viruses types A and B, parainfluenza viruses types 1-4, parechovirus, RSV[q] types A and B, and rhinovirus). Multivariable regression also proved temperature ≥38.0 ˚C (OR 2.3, 95% CI 1.4-3.9) and cough (OR 2.4, 95% CI 1.3-4.3) to predict any respiratory virus infection in febrile children (as stated by parents) with a low discriminative ability (area under the curve 0.64; 95% CI 0.58-0.71). In multivariate regression analysis, cough was no significant predictor of adenovirus and RSV infection.

Bloom et al [50], 2002	United States	Prospective case series study	86 patients presenting with RTI (mean age 20.3 y, 55% female; 111 illness episodes), 1 family practice	URTI[r]	Positive culture for potential pathogens of respiratory tract infection was associated with "purulent discharge from any site" or a "red swollen eye." "Cough," "fever," "sputum" and 9 other signs without significant association to positive culture.
Nakanishi et al [51], 2010	Japan	Retrospec-tive study	406 patients aged <75 y (no gender information) [49] with symptoms of LRTI, emergency department of 1 outpatient clinic	CAP[s]	In the multivariate analysis of clinical symptoms and signs, "cough" was no significant predictor of CAP (patients with CAP and cough n=158), patients without CAP and cough (n=228, OR 1.002, 95% CI 0.101-9.982, *P*=.971).
Hopstaken et al [52], 2003	Netherlands	Cross-sectional study	246 patients ≥18 y (no gender information) presenting with symptoms of LRTI, 25 GPs from 15 practices	Radiologically confirmed pneumonia	Univariate analysis (n=243) showed that "cough <2 days" predicted pneumonia (OR 3.8, 95% CI 1.0-13.8, *P*<.05; PPV 36.4, NPV 86.9), whereas "dry cough" was no statistically significant predictor (OR 2.2, 95% CI 1.0–4.7; as 1.0 was rounded up from a smaller number; PPV 20.7, NPV 89.2); in multivariable analysis, the variables "dry cough" (OR 2.77, 95% CI 1.19-6.44), "diarrhea" (OR 5.90, 95% CI 1.89-8.49), and "temperature >38 °C" (OR 3.08, 95% CI 1.35-7.02) were statistically significant predictors of pneumonia. Laboratory diagnostic parameters of erythrocyte sedimentation rate and C-reactive protein proved to have higher ORs and increased the diagnostic accuracy of the symptoms; these results are not

					represented here due to our symptom-related analysis.
Schneider et al [53], 2021	Germany	Prospective diagnostic study	1141 patients (53% female), average age 42.5 (SD 16.4) y presenting for COVID-19 testing (SD 16.4), 19 GPs	COVID-19	Multivariable logistic regression showed highest associations with SARS-CoV-2 infection for “contact with infected person” (OR 9.22, 95% CI 5.61-15.41), “anosmia/ageusia” (OR 8.79, 95% CI 4.89-15.95), “fever” (OR 4.25, 95% CI 2.56-7.09), and “sudden disease onset” (OR 2.52, 95% CI 1.55-4.14) whereas “dry cough” (OR 1.69, 95% CI 1.08-2.62) and “limb pain” (OR 1.72, 95% CI 1.02-2.91) were more weakly associated. Diagnostic measures of the questionnaire items and selected decision rules for dry cough: Sens 0.43 (0.36-0.51), Spec 0.69 (0.65-0.71), PPV 0.21 (0.17-0.25), NPV 0.86 (0.84-0.89), LR + 1.38 (1.14-1.67), LR - 0.83 (0.72-0.94), and diagnostic OR 1.67 (1.21-2.31).
Hippisley-Cox et al [54], 2011	United Kingdom	Prospective cohort study	2,406,127 derivation cohort (49.9% male); 1,267,151 validation cohort (49.9% male), 30-84 y, 564 general practices using a large primary care database (375 derivation arm; 186 validation arm)	BC[t]	Identified risk factors for newly diagnosed BC in the following 2 years (adjusted HR[u] [95% CI]) for female and male were as follows: “current hemoptysis” (female: 23.9 [20.6-27.6; male: 21.5 [19.3-23.9]), “new onset cough in last 12 months” (female: 1.90 [1.56-2.32]; male: 1.47 [1.23-1.75]), “current appetite loss,” “current weight loss,” “anemia,” “smoking status,” “COPD,” “deprivation.” For female individuals also “prior other cancer” was an independent risk factor. Sens and PPV for the diagnosis of BC in

					the next 2 years for “hemoptysis” were as follows: 23.0 (21-24.8) and 6.4 (5.9-7.0) and for the combination of “age ≥ 40y,” “hemoptysis,” and “current or ex-smoker”: 18.4 (16.8-20.1) and 9.7 (8.9-10.7).
Hamilton et al [55], 2005	United Kingdom	Population-based case-control study	247 patients ≥40 y (69% male) with diagnosis of primary lung cancer from 1998-2002 identified via the cancer registry in the population of one city, 1235 controls (69% male), 21 GPs	Lung cancer	Analysis of the primary care records 2 years before diagnosis of lung cancer: cases (n=247) and controls (n=1,235) were compared for frequencies of symptoms. Cases had higher frequencies of hemoptysis (50 [20%] vs 19 [1.5%] of controls; LR 13 [95% CI 7.9-22]), increasing likelihood ratios with rising number of those consulting with cough (first attendance with cough: 106 [43%] vs 166 [13%]; LR 3.2 [95% CI 2.6-3.9], third attendance (69 [28%] vs 82 [6.6%]; LR 4.2 [95% CI 3.2-5.6]; in multivariable analysis, hemoptysis (OR 32, [95% CI 13-81], *P*<.001) and “second attendance with cough” (OR 2.7 [95% CI 1.7-4.4], *P*<.001) among others were significant predictors of lung cancer, hemoptysis remaining significant after exclusion of the final 180 days before diagnosis.

Iyen-Omofoman et al [56], 2013	United Kingdom	Retrospective case-control study	12,074 patients (59.25% male) with incident diagnosis of lung cancer ≥40 y, 120,731 controls (48.07% male), using a large nationally representative database of general practice records in the United Kingdom	Lung cancer	Comparison of cases and controls in the 4-12 and 13-24 mo periods before lung cancer diagnosis revealed that cases among other factors significantly scored higher for cough (4-12 months prior diagnosis: 1938 (16.05%) vs 7088 (5.87%), unadjusted OR for lung cancer 3.07 (95% CI 2.90-3.24, *P*<.001; 13-24 months prior diagnosis: 1774 (14.69%) vs 9087 (7.53%), OR 2.12 (95% CI 2.00-2.24), *P*<.001) and hemoptysis (4-12 months prior diagnosis: 247 (2.05%) vs. 125 (0.10%), OR 20.15 (95% CI 16.24-25.01), *P*<.001; 13-24 months prior diagnosis: 133 (1.10%) vs 191 (0.16%), OR 7.03 (95% CI 5.63-8.78), *P*<.001). In the multivariate model of factors associated with lung cancer 4-12 months before diagnosis, cough and hemoptysis remained statistically significant predictors (cough: OR 1.63 (95% CI 1.53-1.75), *P*<.001; hemoptysis: OR 8.70 (95% CI 6.75-11.20), *P*<.001). Analysis of sensitivity and specificity of individual symptoms was added in validation population (n=1,826,293). Results for hemoptysis were Sens 1.39, Spec 99.90; for cough: Sens 23.90, Spec 90.43.

Erkens et al [57], 2014	Netherlands	Secondary analysis of a prospective cohort study	516 patients (72% female) with suspected pulmonary embolism which was then excluded, 17-91 y, >300 primary care doctors	Relevant differential diagnoses of pulmonary embolism	Patients diagnosed with a clinically relevant disease[v] were more likely to have presented with a "heart rate of >100 beats per minute (BPM)" (OR 5.1, (95% CI 3.1-8.3), LR+ 3.69 (2.50-5.46), LR- 0.72 (0.64-0.82)), "hemoptysis" (OR 3.3 (1.2-9.0); LR+ 3.17 (1.20-8.35); LR- 0.96 (0.92-1.00)), "unexplained cough" (OR 2.0 (1.3-3.0); LR+ 1.58 (1.23-2.04); LR- 0.79 (0.68-0.92)) or an "unexplained (sudden) onset of dyspnea" (OR 1.6 (1.1-2.4); LR+ 1.23 (1.05-1.43); LR- 0.76 (0.60-0.96)).
Wallander et al [58], 2007	United Kingdom	Prospective cohort study	6913 patients (dyspepsia cohort), 11,036 patients (no gender information), control cohort, 20-79 y, data from 1 GP research database	Dyspepsia	No association of new diagnosis of dyspepsia with "pre-existing asthma," "COPD," "cough" (OR 1.1 (95% CI 1.0-1.3)), "otitis," "laryngitis," "pneumonia" or "stress." "Cough" was the most frequent new diagnosis in both cohorts during the year after index date, but patients with dyspepsia carried an increased risk (7.4%) compared with the control cohort (4.7%) (OR 1.5 (1.3-1.7)).
Hollenz et al [59], 2002	Germany	Prospective study	162 patients (57% female), > 18 y, without known gastroesophageal reflux disease, 1 GP	Gastroesophageal reflux disease (GERD)	Patients with GERD (n=82) compared to those without GERD (n=80) were affected more often from "hoarseness" (n=24 vs n=9; OR 3.3 (95% CI 1.4-7.6); *P*=.006) and "coughing" (n=34 vs n=13; 3.7 (1.7-7.6); *P*<.001). For nonsmokers (n=123) the differences remained significant ("hoarseness": n=22 vs n=7; 3.4 (1.3-8.7) *P*=.01 and coughing n=25 vs n=7; 4.2 (1.6-10.5) *P*=.002).

Visser et al [60], 1996	Netherlands	Population-based case-control study	1458 patients (62% female) with incident coughing and 4182 controls (62% female) (all >20 y) from 10 GPs	Adverse effect of ACE[x] inhibitor	Cases with incident coughing were more likely than controls to have medication with ACE inhibitors (OR 2.1 (95% CI 1.5-3.1)). The adjusted results (age, gender, number of prescriptions, number of consultations, smoking status, use of nonsteroidal anti-inflammatory drug, β-blockers, thiazides or calcium-antagonists) were no longer significant (OR 1.4 (95% CI 0.9-2.1). Analysis of the individual substances only showed statistically significant results for enalapril (crude: OR 2.6 (95% CI 1.6-4.2); adjusted: OR 1.7 (95% CI 1.03-2.8)) whereas the results for captopril, lisinopril and perindopril were not statistically significant.<br>Significant associations were identified for the duration of exposure < 2 mo (unadjusted: OR 4.8 (95% CI 1.7-13.3); adjusted: OR 3.0 (95% CI 1.05-8.3)). For the exposure of 2-6 months, unadjusted results were statistically significant (OR 2.0 (95% CI 1.1-3.8)) but not the adjusted results (OR 1.2 (95% CI 0.6-2.3)). The exposure of more than 6 months obtained no statistically significant results (unadjusted and adjusted).

[a]GPs: general practitioners.

[b]COPD: chronic obstructive pulmonary disease.

[c]OR: odds ratio.

[d]ROC: receiver operating characteristic.

[e]PPV: positive predictive value.

[f]Sens: sensitivity.

[g]Spec: specificity.
[h]NPV: negative predictive value.
[i]LRTI: lower respiratory tract infection
[j]LR: likelihood ratio.
[k]IgE: immunoglobulin E
[l]OAD: obstructive airway disease.
[m]ARTI: acute respiratory tract infection.
[n]ICHPPC: International Classification of Health Problems in Primary Care.
[o]ILI: influenza-like illness.
[p]RTI: respiratory tract infection.
[q]RSV: respiratory syncytial viruses.
[r]URTI: upper respiratory tract infection.
[s]CAP: community-acquired pneumonia.
[t]BC: bronchial carcinoma.
[u]HR: hazard ratio.
[v]Clinically relevant disease: pneumonia, asthma/COPD, respiratory tract infection, heart failure, pericarditis, lung cancer, other.
[x]ACE: angiotensin-converting enzyme.
